# Bacteriocin Production by Beta-Hemolytic Streptococci

**DOI:** 10.3390/pathogens10070867

**Published:** 2021-07-09

**Authors:** Verena Vogel, Barbara Spellerberg

**Affiliations:** Institute of Medical Microbiology and Hygiene, Ulm University Hospital, Albert-Einstein-Allee 23, 89081 Ulm, Germany; verena-1.vogel@uni-ulm.de

**Keywords:** bacteriocin, *Streptococcus pyogenes*, *Streptococcus agalactiae*, *Streptococcus dysgalactiae* subsp. *equisimilis*, quorum sensing, autoinduction, regulation

## Abstract

Beta-hemolytic streptococci cause a variety of infectious diseases associated with high morbidity and mortality. A key factor for successful infection is host colonization, which can be difficult in a multispecies environment. Secreting bacteriocins can be beneficial during this process. Bacteriocins are small, ribosomally produced, antimicrobial peptides produced by bacteria to inhibit the growth of other, typically closely related, bacteria. In this systematic review, bacteriocin production and regulation of beta-hemolytic streptococci was surveyed. While *Streptococcus pyogenes* produces eight different bacteriocins (Streptococcin A-FF22/A-M49, Streptin, Salivaricin A, SpbMN, Blp1, Blp2, Streptococcin A-M57), only one bacteriocin of *Streptococcus agalactiae* (Agalacticin = Nisin P) and one of *Streptococcus dysgalactiae* subsp. *equisimilis* (Dysgalacticin) has been described. Expression of class I bacteriocins is regulated by a two-component system, typically with autoinduction by the bacteriocin itself. In contrast, a separate quorum sensing system regulates expression of class II bacteriocins. Both identified class III bacteriocins are plasmid-encoded and regulation has not been elucidated.

## 1. Introduction

Bacteriocins are small antimicrobial peptides or proteins of bacterial origin. They inhibit the growth of other, often closely related, bacteria, resulting in a colonization advantage of the producer [1]. The mostly cationic nature of these peptides leads to an efficient interaction with the negatively charged bacterial membrane, enabling membrane permeabilization, the main mechanism of action. Typical bacteriocin features like high thermal and pH stability in connection with antimicrobial activity provide interesting characteristics for an application in food preservation. Due to their antimicrobial effects, bacteriocins are also investigated in clinical settings to explore their potential as alternative therapeutics for infectious diseases [2]. Other clinical applications of bacteriocins include oral care, treatment of peptic ulcers and skin care [3]. Additionally, bacteriocins are applied in food preservation and plant growth promotion. Bacteriocins of Gram-positive bacterial species are subdivided into four distinct classes, with each class having different characteristics [4]. Class I bacteriocins are small, heat-stable peptides. The mature peptides are formed by post-translational modifications that generate non-proteinogenic amino acids such as lanthionine, β-methyl lanthionine, and dehydrated amino acids [5]. Class II bacteriocins are typically smaller than 10 kDa and rarely harbor any post-translational modifications [6]. Bacteriocins belonging to class III are heat-labile proteins with a molecular weight of around 30 kDa. Class I and II bacteriocins typically cause bacterial membrane permeabilization via direct interactions, whereas some class III bacteriocins can also exert enzymatic functions, such as the degradation of cell wall structures [7]. Class IV bacteriocins are large complex proteins incorporating either lipid or carbohydrate moieties. However, some publications re-classified these macromolecules as bacteriolysins (hydrolytic polypeptides), leaving only three distinct bacteriocin classes [4,8].

Regulation of bacteriocin expression is often dependent on cell density [9,10], which is monitored by quorum sensing (QS) systems to control intra- and interspecies communication [11]. Signaling peptides of QS systems are peptide pheromones or autoinducing peptides that are secreted into the extracellular environment. At the threshold concentration of the autoinducing peptide bacterial group behavior is triggered, such as the production of antimicrobial peptides. In general, two QS activation pathways exist [9]. One activation pathway involves an autoinducing peptide and a cytoplasmic transcriptional regulator. In this case, the transcriptional cytoplasmic regulator typically belongs to the RRNPP family (Rap, Rgg, NprR, PlcR, and PrgX) and is able to modulate gene expression after a direct interaction with the autoinducing peptide [12]. The second system consists of an autoinducing peptide and a two-component signal transduction system. The autoinducing peptide is exported and sensed by the transmembrane histidine kinase of the two-component system. The response regulator is then phosphorylated and acts as a transcription regulator, ultimately leading to an alteration in gene expression. A prominent example of such a system is the bacteriocin-like peptide (*blp*) region in *Streptococcus pneumoniae.* The peptide pheromone BlpC is exported via the ATP-binding cassette transporter BlpAB and sensed by the histidine kinase BlpH, which phosphorylates the transcription activator BlpR [13]. Phosphorylated BlpR turns on the expression of *blp* and bacteriocin-genes. Secretion of BlpC is induced by the competence-stimulating peptide CSP and couples bacteriocin production to competence [14]. The *blp* locus is highly variable and at least 16 bacteriocin encoding genes have been described in connection to the *blp* locus of *S. pneumoniae* [15,16]. An association of bacteriocin production with the Blp system has also been reported in other streptococci, including *Streptococcus mutans, Streptococcus thermophilus,* and *Streptococcus salivarius* [17,18,19,20].

In regulators of bacteriocin production, cross-reactivity of the autoinducing peptide between different species can occur [21]. For class I bacteriocins, the quorum sensing system is normally part of the bacteriocin gene cluster, and the structural gene of the bacteriocin functions as an autoinducer, as it was described for Nisin [22]. In class II bacteriocins, a separate QS system is typically present, which is induced by a peptide pheromone and controls bacteriocin production. In this review, bacteriocin production and its regulation through quorum sensing systems were surveyed for beta-hemolytic streptococci.

**Figure 1:**
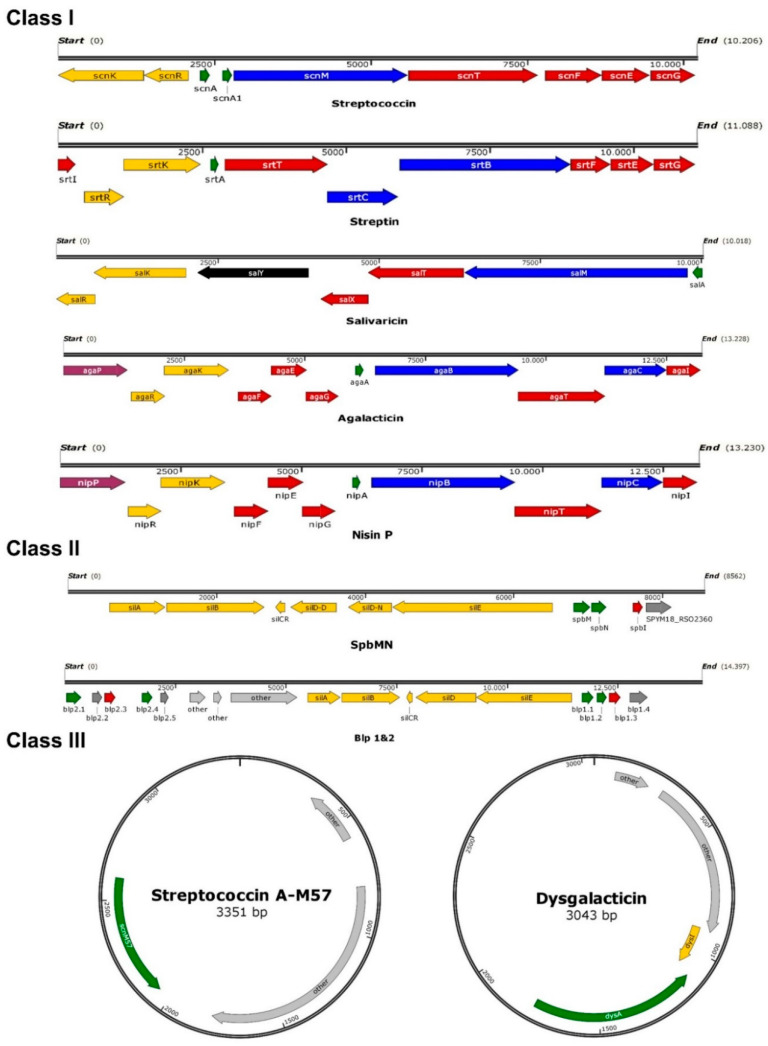
Schematic overview of bacteriocin operons described in beta-hemolytic streptococci. Overview was constructed using SnapGene. Green indicates the structural gene, blue stands for modification, yellow marks regulation genes, genes for transport and immunity are colored red, purple indicates a peptidase, black genes have unknown functions, and grey genes are not part of the bacteriocin gene cluster. To obtain operon organization and DNA sequences of the published bacteriocins, the accession numbers provided by authors were used (Streptococcin A-FF22: AF026542; Streptin: AB030831; Salivaricin A: AE014074; Streptococcin A-M57: AY648561; SpbMN: AE009949; Blp1&2: CP021640; Dysgalacticin: AY907345; Agalacticin: NZ_GL636070; Nisin P: WIDP00000000).

Beta-hemolytic streptococci relevant for human infections mainly comprise *Streptococcus pyogenes* (Group A streptococci, GAS), *Streptococcus agalactiae* (Group B streptococci, GBS), and *Streptococcus dysgalactiae* subsp. *equisimilis* (SDSE). These species cause a variety of infectious diseases and are associated with high levels of morbidity and mortality. Colonization often precedes streptococcal infections and the production of bacteriocins provides a colonization advantage for the producing organism. Understanding the determinants of streptococcal colonization may thus provide the knowledge required for interfering with the development of streptococcal infections. Several bacteriocins of *S. pyogenes* have been described already. In contrast, only very few aspects are known about bacteriocin production in SDSE and *S. agalactiae.* An overview is given in Table 1 and Figure 1.

## 2. *Streptococcus pyogenes*

So far, eight different bacteriocins have been identified and characterized for *S. pyogenes*. Bacteriocin production of *S. pyogenes* was first described in 1971 with the identification of Streptococcin A-FF22 [23]. This 26-amino-acid (aa) peptide is a lantibiotic and belongs to type AII bacteriocins. Type AII lantibiotics are characterized by a ring structure and the typical leader peptide cleavage. Specific genes for modification (*scnM*), transport (*scnT*), immunity (*scnFEG*), and regulation (*scnKR*) of the bacteriocin are present in the A-FF22 gene cluster. Two structural genes with *scnA* and *scnA’* are also part of the *scn* gene cluster [33]. The role of *scnA’* is however unclear since no actual peptide appears to be produced. Pore formation and the subsequent disruption of the membrane potential leads to the death of sensitive target bacteria [34]. Furthermore, the complete gene cluster seems to be part of a mobile genetic element, which may indicate an acquisition of these genes through horizontal gene transfer. The locus can be found in 9% (13 of 144) of *S. pyogenes* strains [35]. In a different *S. pyogenes* background, a minor variant of A-FF22 exists, Streptococcin A-M49. It is encoded by two slightly different structural genes, *scnA’* and *scnA´´*, while the mature bacteriocin A-M49 is identical to mature Streptococcin A-FF22 [36]. *ScnA´* has the same aa sequence as Streptococcin A-FF22, whereas *scnA´´* differs in four aa of the leader peptide sequence. Streptococcin A-M49 is produced by 33% (7 of 21) of M-type 49 strains under anaerobic conditions and shows the same spectrum of antimicrobial activity as A-FF22 [24]. In addition to the sequence similarity of the bacteriocin itself, adjacent genetic regions are identical between Streptococcin A-M49 and Streptococcin A-FF22. A *scnA*-like gene can furthermore be found in *S. salivarius, S. dysgalactiae* and *S. macedonicus.* Regulation of Streptococcin A-FF22 is dependent on a two-component system formed by the response regulator gene *scnR* and the histidine kinase gene *scnK.* An inactivation of one of these genes leads to a loss of Streptococcin A-FF22 production [33]. However, in contrast to other class I bacteriocins, production of Streptococcin A-FF22 does not seem to be autoinducible [35]. A-FF22 has a wide spectrum of antimicrobial activity against Gram-positive species. It inhibits the growth of *Staphylococcus citreus, Micrococcus* spp., *Bacillus* spp., *Corynebacterium* spp., and various streptococci, including *S. pyogenes, Streptococcus. agalactiae, Streptococcus equi*, and *Streptococcus cremoris* [37].

Another lantibiotic, called Streptin, has only been described for *S. pyogenes.* It self-regulates its expression via QS [38]. Two forms of Streptin are known, with Streptin 2 being an unprocessed and differently dehydrated form of Streptin 1. The Streptin gene cluster shows high homology to Nisin and Subtilin gene clusters. *SrtA,* the structural gene for Streptin, is part of a 10-gene operon [25]. Similar to Nisin and Subtilin, the cluster contains genes important for modification (*srtBC*), regulation (*srtRK*), and transport and immunity (*srtT, srtI, srtEFG*). The genes *srtRK* appear to constitute a two-component system that is activated by Streptin [39]. The gene cluster was found to be present in 69% (40 of 58) of *S. pyogenes* strains. However, only 10 of these strains were able to produce active Streptin [38]. In some cases, the *srt* locus showed mutations, but several strains had an intact *srt* locus without producing Streptin. Concerning its spectrum of antibacterial activity, the bacteriocin inhibits the growth of *S. pyogenes* strains and *Micrococcus luteus.*

A third lantibiotic expressed by *S. pyogenes* is Salivaricin A. It is a type IIA lantibiotic that was first described in *S. salivarius*, where it can be found in approximately 10% of the strains [40]. In *S. pyogenes*, Salivaricin A seems to be exclusively expressed by M4-isolates, even though the majority of analyzed strains contain the *sal* locus [21,41]. The production of Salivaricin A has been reported for at least six different streptococcal species, among them *S. dysgalactiae* and *S. agalactiae* [41]. Furthermore, five different variants have been described (*salA1-5*) [41]. Expression of *salA* is autoinduced and all genes necessary for quorum sensing (*salTKR*) as well as for modification (*salM*) and immunity (*salXY*) are part of one gene cluster [11,29,30]. Autoinduction capacity is not limited to certain subtypes, in fact, all subtypes are able to induce the production of the other subtypes [21]. Even though a weak interaction of Salivaricin A2 with lipid II exists, the mechanism of action has not been elucidated [42]. Salivaricin A of *S. salivarius* is active against *S. pyogenes, M. luteus*, *S. pneumoniae* and *Corynebacterium* spp. [42]. To our knowledge, the activity of Salivaricin A produced by *S. pyogenes* has not been investigated.

In contrast to class I bacteriocins of *S. pyogenes*, class II bacteriocins are regulated by an independent quorum sensing mechanism. In this context, only the *streptococcal invasion locus (sil)* has been described. The *sil* locus was first identified by Hidalgo-Grass and has been demonstrated to be important for the virulence of *S. pyogenes* [43]. Furthermore, this locus shows high homologies to the competence system of *S. pneumoniae,* which is linked to bacteriocin production [13,43]. The *sil* locus consists of a two-component system (SilAB), an autoinducing peptide (SilCR), and an ATP-binding cassette transporter system (SilDE) [43,44]. The histidine kinase SilB senses extracellular SilCR and phosphorylates the response regulator SilA. Phosphorylated SilA alters gene expression and induces SilCR production, which is subsequently processed and excreted by SilDE.

While 16% of clinical *S. pyogenes* isolates contain a *sil* system, only 9% seem to have a functional system, since the other strains carry relevant mutations of this locus [45]. One possible explanation for this finding may be that *sil* originally provided an advantage to *S. pyogenes* strains for survival in a polymicrobial environment, but degenerated during adaptation to the host [27]. However, even possessing parts of *sil* may be beneficial. Strains carrying an incomplete *sil* locus can still produce immunity proteins, which provide protection against bacteriocins of competitor strains. Essential in this context is an intact sensing system (SilAB) enabling a reaction to SilCR present in the environment.

Adjacent to the *sil* region, two class II bacteriocins have been described. Armstrong et al. investigated the bacteriocin *(spb)* genes of *S. pyogenes* strain MGAS8232 [26]. In this strain, however, the *sil* system does not seem to be functional. *SpbM* and *spbN* encode putative bacteriocins, which share homologies to *blpMN* of *S. pneumoniae*. Both peptides have a double-glycine-leader peptide, which is necessary for export of the mature peptide [46]. Furthermore, SpbM and SpbN contain GxxxG motifs. These motifs are important for the interaction of the antimicrobial peptides with each other and furthermore for insertion into the bacterial membrane of sensitive strains [6]. When tested in a deferred antagonism assay, only the combination of SpbM and SpbN was able to inhibit target strains, establishing that SpbMN is a two-component bacteriocin. Antimicrobial activity was demonstrated for several *S. pyogenes* strains as well as for *Streptococcus dysgalactiae*, *Streptococcus uberis*, *Micrococcus luteus,* and *Lactococcus lactis*.

A second and third class II bacteriocin encoded by a genetic locus adjacent to *sil* were described by Hertzog et al., Blp1 and Blp2 [27]. The study investigated *S. pyogenes* strain JS12, which, in contrast to other *S. pyogenes* strains, encodes a functional *sil* system. Hallmark traits of class II bacteriocins including a double-glycine-leader peptide and a GxxxG motif can be detected in the peptides Blp1.1 and Blp1.2 that are encoded in the *blp1* region. These two peptides work as a two-component bacteriocin (Blp1). Another functional bacteriocin Blp2 is encoded in the *blp2* region. Both of these bacteriocins are produced by the strain J12 and inhibit other *S. pyogenes* strains. Analyzing publicly available genomes of *S. pyogenes* strains harboring a *sil* system revealed that putative bacteriocin genes are commonly found adjacent to *sil*. Bioinformatic tools are available to efficiently identify and characterize these bacteriocins [47].

A class III bacteriocin designated Streptococcin A-M57 is exclusively produced by M-type 57 *S. pyogenes* strains [24,36,48]. Streptococcin A-M57 is plasmid-encoded and sensitive towards heat and proteases. It does not inhibit the growth of other *S. pyogenes* strains but targets several other Gram-positive bacteria such as *Micrococcus luteus*, *Lactococcus lactis*, *Listeria* spp., *Bacillus megaterium,* and *Staphylococcus simulans*. Due to structural similarities of Streptococcin A-M57 to Dysgalacticin (see SDSE), a similar mode of action, including impaired glucose uptake and disturbed membrane potential, is proposed.

## 3. *Streptococcus dysgalactiae* Subspecies *equisimilis*

While the *sil* locus was first described in *S. pyogenes*, it is much more prevalent in SDSE with 82% of the strains harboring this genetic region [49]. Like in *S. pyogenes*, a class II bacteriocin *blpM* gene cluster can be found adjacent to this locus. However, the functionality of these genes has not been investigated. Nevertheless, at a descriptive level, there are reports of SDSE showing inhibitory activity against other streptococci and *Corynebacterium* spp., but no link to known bacteriocins or bacteriocin genes has been established [50,51,52].

One bacteriocin of SDSE has been characterized at a functional level. It is Dysgalacticin, which belongs to the class III bacteriocins and shows similarities in secondary structure to Streptococcin A-M57 of *S. pyogenes.* It is a 21.5kDa large protein encoded on an indigenous plasmid [29]. Of its 220 aa, the first 28 aa seem to be a leader peptide, causing an export via the *sec* dependent pathway, as described for Zoocin A [53].

Dysgalacticin was shown to target the phosphoenolpyruvate-dependent glucose and mannose phosphotransferase system (PTS) of *S. pyogenes*, leading to a starvation of the bacterial cell [30]. Furthermore, Dysgalacticin causes a disturbed membrane potential and the increased membrane permeability leads to a leakage of intracellular ions. Adjacent to the structural gene *dysA*, the gene *dysI,* encoding an immunity factor, is found [54]. DysI is a membrane-associated protein, protecting producing cells from the detrimental effects of Dysgalacticin by a putative interaction with the glucose and mannose PTS, which constitutes the Dysgalacticin receptor of the target cell. The regulation of Dysgalacticin production in SDSE has not been explored. In contrast to other streptococcal bacteriocins, Dysgalacticin has a narrow spectrum of activity that appears to be limited to *S. pyogenes* strains.

## 4. *Streptococcus agalactiae*

In 1983, the production of antimicrobial inhibitory substances was described for Group B streptococcal strains, isolated from humans and animals [51]. All in all, only 5% of the *S. agalactiae* isolates showed any inhibition of other bacteria. However, characteristics of a class III bacteriocin (large, heat-labile protein) and of a small, heat-resistant bacteriocin were described, but in the years following the initial observation, no further molecular genetic studies were reported on these findings.

Just recently, a first detailed molecular description of a bacteriocin was provided for *S. agalactiae* [31]. Agalacticin was identified by genome analysis with the data mining tool BAGEL3 and is produced by the *S. agalactiae*-type strain ATCC 13813 [31]. It shows high similarity to Nisin, including the leader peptide. All genes necessary for modification (*agaBC*), transport (*agaT*), immunity (*agaI* and *agaFEG*), regulation (*agaRK*), and leader peptidase (*agaP*) are present within the gene cluster. Agalacticin shows antimicrobial activity against several Gram-positive species (*Enterococcus faecalis, Bacillus cereus, Staphylococcus aureus MRSA, Micrococcus flavus*, and *Listeria monocytogenes*). However, regulation of the expression of Agalacticin has not yet been investigated. The similarity to the Nisin gene cluster as well as the presence of *lanRK,* which encodes a putative two-component regulatory system, point in the direction of a quorum sensing system autoregulated by Agalacticin. Another bacteriocin described for *S. agalactiae* is Nisin P, which was identified in *S. agalactiae* strain DPC7040 [32]. Nisin P production required an initial induction with Nisin A but, over time, self-induction occurred. Additionally, a cross reactivity of the Nisin A promotor to Nisin P was observed, even though higher concentrations of Nisin P are needed to activate the promotor. Several Gram-positive species, especially lactobacilli and staphylococci, were inhibited by Nisin P. Since peptide sequence, operon organization and the size of Agalacticin and Nisin P are identical, the same peptide may have been described in both publications under different names. Furthermore, DNA sequence analysis revealed 99.96% of sequence identity.

## 5. Conclusions

Beta-hemolytic streptococcal species are part of polymicrobial environments that require competing with the commensal microbiota. Interestingly, while for *S. pyogenes* a variety of bacteriocins have been described, only a few are reported for SDSE and *S. agalactiae.* The identified bacteriocins are mainly active against other Gram-positive bacteria and closely related streptococcal species, providing a colonization advantage to bacteriocin producers. Understanding the regulation of these peptides might help in elucidating the role they play in microbial communities and their importance for host colonization.

## Figures and Tables

**Table 1:** Schematic overview of bacteriocins produced by beta-hemolytic streptococci. N.D. indicates not determined.

Bacterial Species	Bacteriocin	Class	Proposed Mechanism	Regulation	Antimicrobial Spectrum	Presence of Bacteriocin Locus	Reference
*S. pyogenes*	Streptococcin A-FF22/A-M49	Class I	Membrane permeabilization	Two-component system	*S. pyogenes, S. agalactiae, S. equi, S. cremoris, Micrococci, Bacillus* spp., *Corynebacterium* spp., *S. citreus*	9% of 144 strains; 33% of 21 tested M49 strains	[23,24]
*S. pyogenes*	Streptin	Class I	N.D.	Two-component system	*S. pyogenes, M. luteus*	67% have the *srt* locus with 17% active expression	[25]
*S. pyogenes*	Salivaricin A	Class I	Weak interaction with lipid II (assumed: inhibition of cell wall synthesis and membrane permeabilization)	Two-component system	N.D.*	86% of 144 strains	[21]
*S. pyogenes*	SpbMN	Class II	Two-component bacteriocins (assumed: pore formation)	*Streptococcal invasion locus* (independent QS)	*S. pyogenes, S. dysgalactiae*, *Streptococcus uberis*, *M. luteus, Lactococcus lactis*.	16% of contain a functional *sil* locus	[26]
*S. pyogenes*	Blp1	Class II	Two-component bacteriocins (assumed: pore formation)	*Streptococcal invasion locus* (independent QS	*S. pyogenes*	16% of contain a functional *sil* locus	[27]
*S. pyogenes*	Blp2	Class II	N.D.	*Streptococcal invasion locus* (independent QS	*S. pyogenes*	16% of contain a functional *sil* locus	[27]
*S. pyogenes*	Streptococcin A-M57	Class III	Assumed: Disruption of membrane potential, impaired glucose uptake, increased membrane permeability	N.D.	*M. luteus*, *L. lactis*, *Bacillus megaterium, Staphylococcus simulans, Listeria* spp.,	M-type 57 isolates	[28]
SDSE	Dysgalacticin	Class III	Disruption of membrane potential, impaired glucose uptake, increased membrane permeability	N.D.	*S. pyogenes*	N.D.	[29,30]
*S. agalactiae*	Agalacticin	Class I	Assumed: Nisin-like mechanism (inhibition of cell wall synthesis and membrane permeabilization)	Two-component system	*Enterococcus faecalis, Bacillus cereus, Staphylococcus aureus MRSA, Micrococcus flavus* and *Listeria monocytogenes*	N.D.	[31]
*S. agalactiae*	Nisin P	Class I	Assumed: Nisin-like mechanism (inhibition of cell wall synthesis and membrane permeabilization)	Two-component system with autoinduction	*Lactobacillus* spp., *Staphylococcus* spp.	N.D.	[32]

* Antimicrobial spectrum determined for Salivaricin A of *S. salivarius* includes *Corynebacterium* spp., M. luteus, *S. pyogenes* and *Streptococcus pneumoniae*.

## Data Availability

Not applicable.
